# Effectiveness of registered dietitian-led management of early nutritional support in the emergency intensive care unit: a retrospective observational study

**DOI:** 10.1186/s40795-024-00904-3

**Published:** 2024-07-05

**Authors:** Mamoru Hayashi, Yuki Nishikido, Hiroyuki Banno, Tsuzuki Michitaka, Eiko Tachibana, Takayoshi Tsukahara

**Affiliations:** 1 Nutrition Section, Department of Medical Technology, Japanese Red Cross Aichi Medical Center Nagoya Daiichi Hospital, 3-35, Michishita-Cho, Nakamura-Ku, Nagoya, Aichi 453-8511 Japan; 2 Graduate School of Nutrition Science, Nagoya University of Arts and Sciences, Aichi, Japan; 3 Nursing Department, Japanese Red Cross Aichi Medical Center Nagoya Daiichi Hospital, Aichi, Japan; 4 Intensive Care and Emergency Department, Japanese Red Cross Aichi Medical Center Nagoya Daiichi Hospital, Aichi, Japan

**Keywords:** Nutritional support, Enteral nutrition, Critical illness, Intensive care unit, Oral feeding, Tube feeding

## Abstract

**Background:**

Appropriate nutritional management in critically ill patients positively impacts prognosis. This study evaluated the effectiveness of a dietitian-led early enteral nutrition protocol in an intensive care unit (ICU).

**Methods:**

This retrospective analysis of prospectively collected data included patients who stayed in the emergency ICU (EICU) for at least 5 days between April 2021 and May 2022. Patients were divided into control and early support groups based on the implementation of the early enteral nutrition protocol in November 2021.

**Results:**

The time to start enteral nutrition after admission was significantly shorter in the early support group (41.9 h) than in the control group (59.8 h). The early support group (*n* = 58) also had higher nutritional sufficiency rates than the control group (*n* = 56) and a lower incidence of diarrhea (10% vs. 37.5%).

**Conclusions:**

The dietitian-led early nutritional support system effectively reduced the time to enteral nutrition initiation, improved nutritional sufficiency rates, and decreased the incidence of diarrhea in the EICU.

## Background

Nutritional therapy aims to prevent or mitigate organ failure, sustain nitrogen balance, and preserve lean body mass by providing an adequate supply of carbohydrates, lipids, proteins, and various nutrients [1, 2]. The fundamental principle underlying this approach is “if the gut is functional, utilize it” [3]. Appropriate nutritional management, including enteral nutrition (EN), yields improved outcomes even in critically ill patients, underscoring its significance [4]. Various randomized controlled trials and meta-analyses have reported reductions in infection rates [5], medical costs [6], length of stay in the intensive care unit (ICU) [7], and number of days on mechanical ventilation [8] associated with EN alone or EN and parenteral nutrition together [9–11]. Early EN (EEN) initiation is crucial for critically ill patients, and the Society of Critical Care Medicine (SCCM) and American Society for Parenteral and Enteral Nutrition (A.S.P.E.N.) guidelines recommend starting EN within 24–48 h of ICU admission to improve clinical outcomes, which has been associated with reduced complications and improved delivery rates of EN [1, 2]. However, without a dedicated ICU dietitian, the initiation and adherence to EN protocols can be inconsistent, leading to delays in nutritional support and increased incidence of complications such as infections and gastrointestinal issues [4].

The Japanese Guidelines for Nutritional Therapy of Critically Ill Patients (hereinafter referred to as “the guidelines”) strongly advocate the prioritization of EN as the preferred route for nutritional administration [12]. A noteworthy study reported a reduction in mortality rates among trauma patients requiring intensive care in the intensive care unit (ICU), who had received EN within 48 h of admission [13]. In light of these findings, the guidelines recommend initiating EN within 24 h, with 48 h as the outer limit [11]. Additionally, evidence suggests that total parenteral nutrition results in less favorable postoperative outcomes compared with EEN after major surgeries, such as cystectomy, further emphasizing the need for timely EN initiation [9].

In 2019, the Japanese Red Cross Aichi Medical Center Nagoya Daiichi Hospital (hereinafter referred to as “the hospital”) initiated multidisciplinary ICU rounds involving physicians, nurses, pharmacists, physical therapists, clinical engineers, and dietitians. However, the dietitian was not a full-time staff member and participated irregularly. This absence of a full-time dietitian often resulted in delayed initiation of EN and lower adherence to nutritional protocols, which could increase the risk of complications.

The aim of this study was to assess the impact of a full-time, dietitian-led nutrition support system by comparing the nutritional management status, safety, and outcomes of patients before and after the introduction of such an enhanced system in an emergency ICU (EICU).

## Methods

### Study design

This retrospective analysis of prospectively collected data included patients aged above 18 years who were admitted to our EICU for at least 5 days between April 2021 and May 2022. The inclusion criteria were restricted to patients whose data could be obtained for 5 consecutive days during their stay in the EICU. Patients with COVID-19, post-gastrointestinal surgery patients, patients not eligible for aggressive treatment, patients requiring reoperation post-admission, and patients who died within less than 5 days of admission were excluded from the study.

In November 2021, a full-time dietitian was assigned to our EICU. This event marked a significant transition from intermittent involvement to consistent, full-time engagement, enabling the dietitian to intervene more promptly. Moreover, under the dietitian’s leadership, a nutrition initiation flowchart was created to reduce the time to the initiation of EN, and an EN protocol was established to enhance the nutritional sufficiency rate. As part of the protocol, the method of EN administration was changed from intermittent to continuous feeding at slower rates, which may have influenced the incidence of diarrhea.

**Fig. 1:**
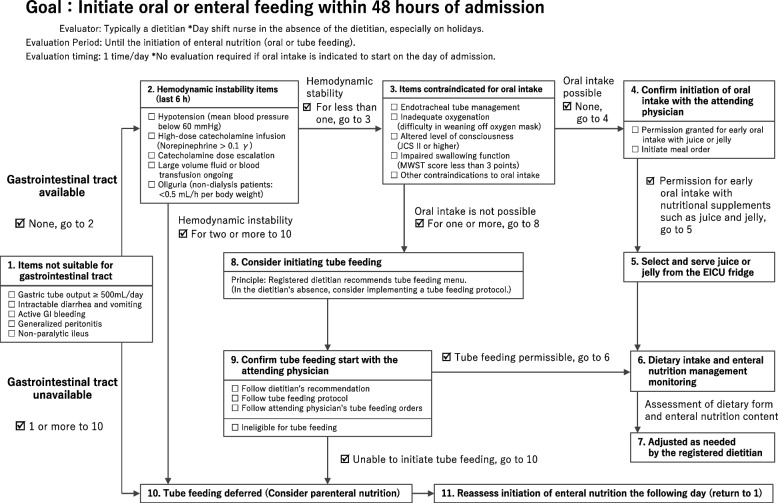
Early nutrition initiation flowchart

The early nutritional support system, which assigns a dedicated dietitian to the EICU, was implemented in November 2021. Patients admitted between April and October 2021 (i.e., before the system was initiated) constituted the control group, whereas those admitted between November 2021 and May 2022 (i.e., after the system was initiated) formed the early support group. An early nutrition initiation flowchart (Fig. 1) was introduced to shorten the time from ICU admission to EN initiation. In addition, a hospital-specific EEN protocol was developed and implemented. In this study, EN encompassed both oral and tube feeding.

### EICU operational system

The operational system of our EICU adheres to the open ICU model. The final authority for decisions about treatment policies and strategies rests with primary attending physicians representing various medical specialties.

### Nutritional support

The full-time EICU dietitian is available on-site from 9:00 AM to 5:00 PM on weekdays (Mondays through Fridays, excluding weekends and holidays). The dietitian actively participated in multidisciplinary rounds in collaboration with intensivists, nurses, pharmacists, physical therapists, clinical engineers, and fellow dietitians, to perform a comprehensive assessment of the patients’ overall health. Subsequently, individualized nutritional management plans were proposed for attending physicians from various medical departments. These plans included calculations of the estimated energy and protein requirements. Maximizing the use of the gastrointestinal tract as the primary route for nutrient administration was a key consideration.

The early nutrition initiation flowchart, which clarified the presence or absence of gastrointestinal disturbances, such as gastrointestinal tract drainage ≥ 500 mL/day or active gastrointestinal bleeding, and circulatory stability criteria, such as mean blood pressure ≥ 60 mmHg or no increase in catecholamine levels, was used as a reference to determine whether oral intake or tube feeding should be initiated (Fig. 1). In addition, nutritional supplements, such as jellies and juices, were incorporated into the patients’ diet to shorten the time to oral intake and improve the nutritional sufficiency rate.

In the case of EN through tube feeding, the dietitian used the hospital’s proprietary, EEN protocol while designing nutritional management plans that prevented complications. In situations where complications arose or the patient was deemed to be at high risk, the dietitian also recommended the suitable content and administration methods of nutritional supplements and adjustments to pharmacotherapy. Challenges with gastrointestinal tract usage prompted the consideration of intravenous nutrition and associated nutritional administration.

### Evaluation

The primary endpoints of the study were the time that elapsed from EICU admission to EN initiation and the percentage of patients who initiated EN within 48 h of EICU admission. Secondary endpoints included energy and protein sufficiency rates after EN up to 7 days after admission to the EICU, rate of complications such as diarrhea and vomiting during EN management as a measure of gastrointestinal tolerance, duration of hospital stay, duration of stay in the EICU, and patient outcomes (e.g., discharge, transfer to another facility, and in-hospital death).

Energy sufficiency rate (%) was calculated as energy intake (kcal) divided by energy requirement (current body weight (kg) multiplied by 25 kcal) multiplied by 100 on each day from days 1 to 7 after EICU admission for patients with a body mass index (BMI) of less than 30; for patients with a BMI of 30 or greater, energy sufficiency rate (%) was calculated as energy intake (kcal) divided by energy requirement (ideal body weight (kg) multiplied by 25 kcal) multiplied by 100. Protein sufficiency rate (%) was calculated as protein intake (g) divided by protein requirement (current body weight (kg) multiplied by 1.2 g) multiplied by 100 for each day from days 1 to 7 after EICU admission. Diarrhea was defined as the occurrence of loose or watery stools three or more times per day after the start of tube feeding.

Other endpoints included demographic information such as sex, age, height, weight, BMI, serum albumin level, acute physiologic chronic health assessment (APACHE II) score [14], organ failure assessment (SOFA) score [15], and modified nutritional risk in critically ill patients (mNUTRIC) score [16].

### Statistical analysis

The results for each evaluation criterion are presented as either mean ± standard deviation or median (first quartile–third quartile), along with the number of patients. In the comparison of the two groups, the normality of continuous variables was confirmed, and either Student’s t-test (for normally distributed data, e.g., age, height, weight, BMI, serum albumin level, APACHE II score, SOFA score, and mNUTRIC score) or Mann–Whitney U test (for non-normally distributed data, e.g., energy and protein sufficiency rates) was used. For nominal variables, statistical analysis was performed using the chi-square test, and when the expected frequency was less than five, Fisher’s exact test was used instead. To assess and compare the increase in energy and protein sufficiency rates up to day 7 of EICU admission between the groups, a Mann–Whitney U test was initially performed to evaluate the differences in sufficiency rates between the groups each day. We used linear mixed model analysis to investigate the overall increase in energy and protein sufficiency rates over the entire 7-day period. This analysis considered the sufficiency rate as the dependent variable, with the presence or absence of support, duration of ICU stay, and their interactions as independent variables. The analysis included sex, age, BMI, SOFA score, and APACHE II score as covariates. To account for individual variability, we adjusted for individual intercepts and daily slopes, and the correlation structure of intra-individual variation was assumed to be a compound symmetrical correlation structure that remained unaffected by repetitions.

Statistical analysis was conducted using R ver. 4.2.1 (R Foundation for Statistical Computing, Vienna, Austria), and the nlme package was used for the linear mixed model analysis. Statistical significance was set at *P* < 0.05.

## Results

### Study patients

**Fig. 2:**
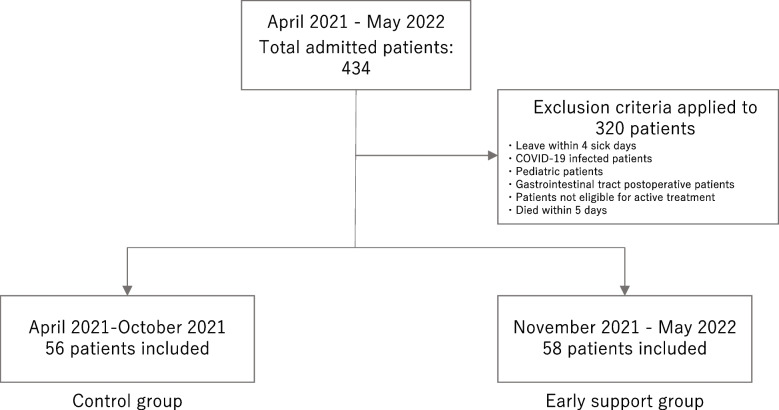
Study patients

During the study period, 434 patients were admitted to the EICU. In total, 320 individuals met the exclusion criteria and were consequently excluded from the study. The control group consisted of 56 individuals, whereas the early support group consisted of 58 individuals (Fig. 2).

Patient demographics at admission are presented in Table 1. The early support group had a significantly lower BMI than the control group (*p* = 0.035). However, no significant differences were found in other parameters between the groups. The distribution of underlying diseases also did not differ significantly between the groups (Table 2).

**Table 1:** Patient demographics at admission

	Contorol group (*n* = 56)	Early support group (*n* = 58)	*p* value
Sex [Male (%)/Female (%)	36 (64.3) / 20 (35.7)	40 (69.0) / 18(31.0)	*0.596
Age	63.7 ± 15.0	67.3 ± 14.6	†0.191
Height(cm)	161.4 ± 9.4	162.4 ± 8.5	†0.564
Body weight(kg)	66.1 ± 19.3	61.4 ± 14.2	†0.144
BMI(kg/m^2^)	25.1 ± 5.6	23.1 ± 4.1	†0.035
Alb(g/dL)	3.7 ± 0.8	3.8 ± 0.7	†0.842
APACHE II score	26.2 ± 8.8	23.3 ± 8.1	†0.075
SOFA score	6.1 ± 3.9	5.1 ± 2.9	†0.129
mNUTRIC score	4.4 ± 2.0	4.2 ± 1.9	†0.674
Ventilator [Yes (%)/No (%)	34 (60.7) / 22 (39.3)	30 (51.7) / 28 (48.3)	*0.352

Mean ± SD

Gender and number of patients with ventilator (%)

*BMI* Body mass index, *APACHE II* Acute physiology and chronic health evaluation II, *SOFA* Sequential organ failure assessment, *mNUTRIC* Modified nutrition risk in the critically ill

^*^Chi-squared test

^†^Student’s t-test

**Table 2:** The distribution of underlying diseases

	Contorol group (*n* = 56)	Early support group (*n* = 58)	*p* value
Cardiology [persons (%)]	25 (44.6)	24 (41.4)	0.355
Neurosurgery [persons (%)]	15 (26.8)	23 (39.7)	
Cardiovascular Surgery [persons (%)]	6 (10.7)	6 (10.3)	
Other [persons (%)]	10 (17.9)	5 (8.6)	

Fisher’s exact test

### Time from EICU admission to EN initiation

The time from admission to the EICU to the initiation of EN was significantly shorter in the early support group at 41.9 (21.8–51.2) h than in the control group at 59.8 (30.6–72.7) h (*p* < 0.001). Furthermore, the rate of EEN initiation within 48 h of EICU admission was significantly higher in the early support group (74.1%) than in the control group (60.7%) (*p* < 0.001) (Table 3).

**Table 3:** The rate of early enteral nutrition initiation within 48 h of EICU admission

	Control group (*n* = 56)	Early support group (*n* = 58)	*p* value
Achievement (%)	34 (60.4)	43 (74.1)	< 0.001
Non-achievement (%)	22 (39.3)	15 (25.9)	

Chi-squared test

### Energy and protein sufficiency rates

**Fig. 3:**
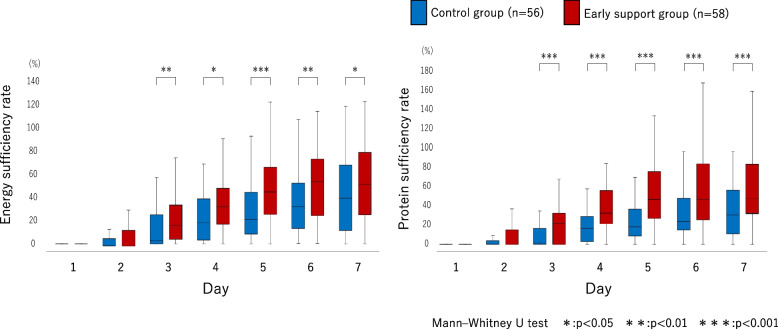
Energy and protein sufficiency rates of enteral nutrition from days 1 to 7 in the EICU. Mann–Whitney U test. *: *p* < 0.05, **: *p* < 0.01, ***: *p* < 0.001. EICU, emergency intensive care unit

The energy and protein sufficiency rates of EN from days 1 to 7 in the EICU were significantly higher in the early support group from days 3 to 7 than in the control group (Fig. 3). Additionally, a linear mixed model analysis of the energy and protein sufficiency rates, adjusted for covariates, including BMI, SOFA score, APACHE II score, age, and sex, revealed a significant interaction between the control and early support groups and duration of ICU stay (Table 4).

**Table 4:** A linear mixed model analysis adjusted for covariates

	Energy	Protein
(Intercept)	-0.00 (0.09)	0.05 (0.10)
Day	0.07 (0.01)^a^	0.06 (0.01)^a^
Early support group	-0.02 (0.03)	-0.05 (0.04)
Day:Early support group	0.02 (0.01)^b^	0.05 (0.01)^a^
Sex(Male)	-0.01 (0.02)	-0.05 (0.03)
Age	0.00 (0.00)	0.00 (0.00)
BMI	-0.00 (0.00)	-0.01 (0.00)^b^
APACHE II score	-0.00 (0.00)	0.00 (0.00)
SOFA score	-0.00 (0.00)	-0.00 (0.00)

Partial regression coefficient (SE)

Dependent Variable: Energy and Protein sufficiency up to day 7 of EICU admission

Fixed Effects: Sex, Age, BMI, APACHE II score, SOFA score

Random Effects: Length of Stay in the EICU

^a^*p* < 0.001, ^b^*p < 0.05*

### Complications in tube feeding management, duration of EICU stay, duration of hospital stay, and outcomes

Regarding the occurrence of complications in tube feeding management up to day 7 in the EICU, the early support group had a significantly lower incidence of diarrhea than the control group (10% vs. 37.5%). The two groups showed no significant differences in the incidence of vomiting, interruptions due to increased residual gastric volume, or interruptions due to deteriorating vital signs (Table 5). Although no significant differences were observed in the duration of EICU stay, duration of hospital stay, or outcomes between the groups, inpatient mortality was lower in the early support group than in the control group (Table 6).

**Table 5:** Complications in tube feeding management up to day 7 in the EICU

		Contorol group (*n* = 56)	Early support group (*n* = 58)	*p* value
Diarrhea	Yes(%)	12 (37.5)	3 (10)	^*^0.017
	No(%)	20 (62.5)	27 (90)	
Vomiting	Yes(%)	8 (25)	3 (10)	^*^0.122
	No(%)	24 (75)	27 (90)	
Interruptions due to increased residual gastric volume	Yes(%)	7 (21.9)	2 (6.7)	^†^0.149
	No(%)	25 (78.1)	28 (93.3)	
Interruptions due to deteriorating vital signs	Yes(%)	4 (12.5)	3 (10)	^†^1.000
	No(%)	28 (87.5)	27 (90)	

^*^Chi-squared test

^†^Fisher’s exact test

**Table 6:** Duration of EICU stay, duration of hospital stay, and outcomes

	Contorol group (*n* = 56)	Early support group (*n* = 58)	*p* value
EICU stay	7.6 ± 2.7	8.3 ± 4.1	^*^0.307
Hospital stay	33.5 ± 22.7	36.4 ± 23.6	^*^0.506
Discharges and separations [person (%)] /in-hospital deaths [person (%)]	45 (80.4) / 11 (19.6)	54 (93.1) / 4 (6.9)	^†^0.055

Mean ± SD

^*^Student’s t-test

^†^Chi-squared test

## Discussion

In this study, we developed an early nutritional support management system by deploying dedicated dietitians in the EICU and implementing an early nutritional initiation flowchart, along with an EEN protocol. Consequently, the time from EICU admission to the commencement of EN was reduced, and the rate of EN initiation within 48 h post-admission increased. Additionally, energy and protein sufficiency rates improved, and the incidence of diarrhea during EN management decreased.

Previous studies have emphasized the importance of dedicated dietitians in ICU settings. Their focus on nutritional management has been associated with a reduced time from ICU admission to the initiation of EN [1, 2]. A study involving 81 facilities worldwide also reported higher rates of EEN initiation in ICUs with dietitians [2]. Furthermore, authorizing dietitians to create nutrition-related orders in acute care hospitals can reduce the time to implement oral nutritional supplementation [4]. Our results align with these findings, as we observed similar improvements by implementing an early nutrition support system immediately after admission to the EICU using a locally developed early nutrition initiation flowchart and enteral feeding protocol under the guidance of a full-time dietitian, although no dedicated dietitian was authorized to write orders. These results highlight the critical role of dietitians in collaborating with multiple healthcare professionals to establish a common understanding of the EN initiation process, which is essential for achieving these outcomes. Recent studies indicate that implementing structured EN protocols can significantly impact clinical outcomes in critically ill patients. For instance, using standardized protocols has been associated with improved nutritional sufficiency rates and reduced complications such as infections and gastrointestinal issues [17, 18]. Our findings align with these observations, highlighting the importance of early and consistent nutritional support in the ICU setting [19]. Moreover, EEN has been linked to better patient outcomes, including lower mortality rates and reduced ICU stay durations [4, 17, 18]. However, the FRANS study indicates that EEN might be associated with higher 28-day mortality rates in critically ill patients [20]. This study found that early nutrition support was more likely to be prescribed to patients with worse conditions, which could generate a strong internal correlation between early nutrition support and higher mortality. Factors such as higher SOFA and Simplified Acute Physiology Score II scores, as well as the use of vasopressors and invasive mechanical ventilation were significantly higher in the early nutrition groups, which might affect the "true" association between early nutrition support and mortality even in adjusted analyses. Therefore, further research is needed to explore optimal protocols and patient-specific factors that can enhance these outcomes.

Regarding the nutritional sufficiency rate, Kim et al. demonstrated that the use of EN protocols leads to improved nutritional sufficiency rates [21]. In our study, we evaluated enteral nutritional intake through both oral and tube feeding. Nutritional sufficiency rates improved even when non-compulsory nutritional administration methods were used. Our analysis using a general linear model revealed that increases in energy and protein sufficiency rates remained significant after adjusting for covariates such as age, sex, BMI, APACHE II score, and SOFA score. These findings suggest that continued nutritional support enhances the nutritional sufficiency rate, attributed to the visualization of daily target feeding quantities through the EEN protocol, the establishment of a shared goal within the multidisciplinary medical team, and increased frequency of dietary adjustments for oral intake by dietitians. This underscores the significant role of dietitians as nutrition specialists in improving nutritional sufficiency through gastrointestinal nutritional support in critical care environments. In a study on nutritional dosage in the ICU, patients were recommended to receive 70% of their estimated energy requirement and a minimum intake of 1.3 g/kg/day of protein by the fourth day of ICU admission [7]. Our early support group did not meet these criteria, which is a challenge to overcome in future studies. Although standardized nutritional management can be achieved through EN protocols, these protocols need to be refined to accommodate variations in clinical characteristics, such as age, body size, and severity of illness.

Furthermore, Qu et al. compared the effects of intermittent versus continuous enteral feeding in critically ill patients through a meta-analysis and reported that intermittent enteral feeding is associated with a higher incidence of diarrhea [17]. In this study, we found a significant decrease in the frequency of diarrhea in the early support group. In contrast, Heffernan et al. compared intermittent versus continuous administration of EN in a systematic review and found no significant differences in the incidence of diarrhea, vomiting, aspiration, or increased gastric residual volume [18].

In our study, patients on EN management received EN through a nasogastric tube. Prior to the introduction of the early nutrition support system, the standard method of administration at the start of EN in our EICU was intermittent administration, which was changed to continuous administration at slower rates after the system was introduced. The change to continuous administration and slower rate of administration were thought to be two of the reasons for the lower incidence of diarrhea and may present as a limitation in interpreting our results related to this outcome. In addition, the type of EN (digestible or semi-digestible), dosage, primary medical condition, severity, nutritional status, and medications also appeared to affect the incidence of complications. Thus, guided by established guidelines, the unique EN protocol at our institution allowed us to enhance the nutritional sufficiency rate without increasing complication rates. Standardizing nutritional management and consistently sharing information improve the efficiency and safety of nutritional care, which is beneficial for patients.

Regarding outcomes, lower in-hospital mortality was observed in the early support group than in the control group. Doig et al. reported a reduction in mortality with EEN in trauma patients requiring intensive care [13]. Similarly, Ortiz-Reyes et al. reported improvements in outcomes during a multicenter study, including decreases in the 28-day mortality rate, number of days spent in the ICU, and duration of ventilator use associated with EEN [10]. However, when the multivariate analysis was adjusted for age, sex, BMI, APACHE II score, and SOFA score, no significant differences in inpatient mortality were observed. Recent studies highlight various factors influencing mortality in critically ill patients, including stress hyperglycemia, comorbid conditions, and organ dysfunction severity. For instance, higher stress hyperglycemia ratios have been linked to increased 28-day mortality in sepsis patients, emphasizing the importance of adjusting for variables such as age, sex, APACHE II scores, and SOFA scores in predictive models [22–25]. Additionally, factors including mechanical ventilation, vasopressor use, blood glucose control, and baseline nutritional status are critical determinants of patient outcomes [24, 25]. These insights emphasize the multifactorial nature of mortality in critically ill patients, which necessitates comprehensive multivariate analyses to accurately interpret study findings. Numerous studies have conclusively demonstrated the benefits of EEN in critically ill patients, including reductions in infection rates, medical costs, length of ICU stay, and duration of mechanical ventilation [22, 23]. However, further research is needed to explore optimal protocols and patient-specific factors that can enhance these outcomes [2, 4].

This study has several limitations. First, the single-center nature of this study limits the generalizability of our findings. Multicenter studies are needed to confirm the applicability of our results in different clinical settings. Additionally, the nutritional management status assessment was limited to the first 7 days after EICU admission, leaving the influence of long-term nutritional therapy unaccounted for. Future studies should consider the long-term effects of nutritional management beyond the initial week of EICU admission. Our study results may also be limited to specific patient populations with certain conditions and severities, and caution should be exercised when interpreting these findings in broader contexts. In the future, it is important to assess how these results apply to a more diverse patient population. Furthermore, we did not evaluate blood glucose levels and their potential impact on patient outcomes. This is significant because studies have highlighted the importance of stress hyperglycemia and its association with increased mortality in critically ill patients. Future studies should include blood glucose monitoring and consider the role of stress hyperglycemia in predicting patient outcomes. Lastly, factors such as mechanical ventilation, vasopressor use, and baseline nutritional status should be comprehensively evaluated to provide a more robust analysis. These factors are critical determinants of patient outcomes and should be included in future research to enhance the validity of the findings.

This study showed that even after adjusting for patient severity and organ damage, the time from ICU admission to the start of EN was significantly shortened, and the nutritional sufficiency rate improved. This suggests the importance of assigning a full-time dietitian to the EICU and strengthening the nutrition management system with the dietitian at the core.

## Conclusions

Enhancing early nutritional support system in the EICU reduced the time to initiate EN and improved nutritional adequacy. This intervention also decreased diarrhea incidence and improved tube feeding safety. Increasing the number of dedicated dietitians will further enhance patient outcomes by enabling better collaborative care. Further research is required to refine protocols and consider patient-specific factors for optimal results.

## Data Availability

No datasets were generated or analysed during the current study.

## Abbreviations

APACHE II: Acute Physiology and Chronic Health Evaluation
BMI: Body mass index
EICU: Emergency intensive care unit
EN: Enteral nutrition
EEN: Early enteral nutrition
ICU: Intensive care unit
mNUTRIC: Modified Nutrition Risk in Critically Ill
SOFA: Sequential Organ Failure Assessment
